# Large-scale antibody immune response mapping of splenic B cells and bone marrow plasma cells in a transgenic mouse model

**DOI:** 10.3389/fimmu.2023.1137069

**Published:** 2023-06-05

**Authors:** Xiaoli Pan, Sheila N. López Acevedo, Camille Cuziol, Evelyn De Tavernier, Ahmed S. Fahad, Priyobarta S. Longjam, Sambasiva P. Rao, David Aguilera-Rodríguez, Mathilde Rezé, Christine A. Bricault, Matías F. Gutiérrez-González, Matheus Oliveira de Souza, Joshua M. DiNapoli, Emmanuelle Vigne, Melody A. Shahsavarian, Brandon J. DeKosky

**Affiliations:** ^1^ Department of Pharmaceutical Chemistry, The University of Kansas, Lawrence, KS, United States; ^2^ Ragon Institute of Massachusetts General Hospital (MGH), Massachusetts Institute of Technology (MIT), and Harvard, Cambridge, MA, United States; ^3^ Department of Chemical Engineering, Massachusetts Institute of Technology, Cambridge, MA, United States; ^4^ Large Molecule Research, Sanofi, Vitry sur Seine, France; ^5^ Large Molecule Research, Sanofi, Ghent, Belgium; ^6^ Large Molecule Research, Sanofi, Cambridge, MA, United States; ^7^ Sanofi Vaccines, Sanofi, Cambridge, MA, United States; ^8^ Department of Chemical Engineering, The University of Kansas, Lawrence, KS, United States

**Keywords:** B cell, antibody discovery, antibody repertoire analysis, yeast surface display, cytokine, spleen, bone marrow, plasma cells

## Abstract

Molecular characterization of antibody immunity and human antibody discovery is mainly carried out using peripheral memory B cells, and occasionally plasmablasts, that express B cell receptors (BCRs) on their cell surface. Despite the importance of plasma cells (PCs) as the dominant source of circulating antibodies in serum, PCs are rarely utilized because they do not express surface BCRs and cannot be analyzed using antigen-based fluorescence-activated cell sorting. Here, we studied the antibodies encoded by the entire mature B cell populations, including PCs, and compared the antibody repertoires of bone marrow and spleen compartments elicited by immunization in a human immunoglobulin transgenic mouse strain. To circumvent prior technical limitations for analysis of plasma cells, we applied single-cell antibody heavy and light chain gene capture from the entire mature B cell repertoires followed by yeast display functional analysis using a cytokine as a model immunogen. We performed affinity-based sorting of antibody yeast display libraries and large-scale next-generation sequencing analyses to follow antibody lineage performance, with experimental validation of 76 monoclonal antibodies against the cytokine antigen that identified three antibodies with exquisite double-digit picomolar binding affinity. We observed that spleen B cell populations generated higher affinity antibodies compared to bone marrow PCs and that antigen-specific splenic B cells had higher average levels of somatic hypermutation. A degree of clonal overlap was also observed between bone marrow and spleen antibody repertoires, indicating common origins of certain clones across lymphoid compartments. These data demonstrate a new capacity to functionally analyze antigen-specific B cell populations of different lymphoid organs, including PCs, for high-affinity antibody discovery and detailed fundamental studies of antibody immunity.

## 1 Introduction

The antibody immune response plays a critical role in adaptive immune protection (1–4). Early B cell development originates from hematopoietic stem cells (HSCs), which differentiate in the bone marrow (BM) to become pro-B cells, pre-B cells, and eventually mature (naïve) B cells (5–7). B cell receptors (BCRs), the membrane-bound form of antibodies, are generated *via* somatic recombination of antibody variable (V), diversity (D), and joining (J) genes for heavy chain variable region (VH), and V, J genes for light chain variable region (VL) (8, 9). Mature B cells leave the bone marrow and migrate through the bloodstream to enter secondary lymphoid organs, such as the spleen (SPL) and lymph nodes, for B cell activation upon antigen encounter. B cell activation induces the formation of germinal centers (GCs) where B cells undergo iterative cycles of somatic hypermutation (SHM) and clonal expansion with constant antigen exposure to create an extremely diverse antibody repertoire and generate high-affinity antibodies targeting a specific antigen through affinity maturation (10–12). After the GC reactions, activated B cells continue to differentiate into different types of effector B cells, including memory B cells and antibody-secreting cells (ASCs) like plasmablasts and plasma cells (PC) (13, 14). B cell development is a thus highly dynamic process that involves multiple lymphoid organs, resulting in highly diverse organ-specific antibody repertoires. Several studies have analyzed clonal overlap among different tissue compartments in mice (15–18), rabbits (19), and humans (20, 21) but rarely have examined antigen-specific B cell functions that could help reveal the origins and distributions of specific functional antibodies across immune compartments.

Over the past decades, single B cell analysis based on fluorescence-activated cell sorting (FACS) has emerged as a dominant tool for both repertoire-scale molecular characterization of antigen-specific human B cell immunity and for the discovery of potent monoclonal antibodies (mAbs) to prevent and treat a wide range of diseases (22–26). B cell discovery campaigns primarily analyze memory B cells and occasionally short-lived plasmablasts, whereas plasma cells (PCs) are rarely employed (27, 28) mainly because of two limitations. First, the majority of plasma cells home to reside in bone marrow after their formation, and it is relatively challenging to obtain human bone marrow tissue samples routinely (13). In addition, plasma cells lack surface BCRs and are thus incompatible with antigen-labeled FACS analysis (29). Despite the technical difficulties, a thorough characterization of PC responses is critical because PCs produce the majority of the circulating antibodies (30–33) and can persist for many years to provide long-term immune protection (30, 34, 35). Plasma cells also carry a higher level of immunoglobulin mRNA than memory B cells and provide the source of antibodies surveyed in rapid and simple ELISA-based serum assays, thus representing an excellent source for antibody discovery (36–38). New approaches are needed to advance functional plasma cell analyses to understand better humoral immune responses across tissue compartments and for PC-based antibody discovery.

Early therapeutic mAb discovery was mainly conducted through mouse immunization with a target antigen followed by hybridoma screening. The generation of chimeric and humanized mAbs largely improved the immunogenicity issues with murine antibodies. However, these engineered antibodies still contain partial murine antibody genes and remain labor-intensive and costly to produce (39–41). The development of phage display technology (42) and transgenic mice (43) further advanced mAb discovery by enabling the direct generation of antibodies from human genes and reducing the need for extensive antibody humanization (44, 45). Compared to *in vitro* phage display screening, the human antibodies generated from transgenic mice go through *in vivo* clonal selection and affinity maturation and appear to have better biophysical attributes and drug-like properties than those isolated from synthetic display libraries (46, 47). Transgenic mice are genetically modified to have the human immunoglobulin (Ig) genes inserted into the mouse genome to replace the endogenous murine Ig genes so that they can produce fully human antibodies upon immunization (40). First-generation transgenic mice incorporated human Ig genes for both variable and constant domains, resulting in low efficiency of human antibody generation, SHM, and class switching due to the lack of murine constant region gene expression (43, 48). Therefore, second-generation transgenic mice like KyMouse, VelocImmune, and Trianni were designed to produce antibodies with human variable regions and murine constant regions that permit optimal signaling for antibody maturation and differentiation (47, 49, 50). Although biased V-gene usage is observed in several transgenic mouse models (49, 51, 52), overall, these animals provide strong opportunities to generate human-like antibodies in response to antigen immunization (53–56).

In this study, we performed functional and molecular characterization of antibody repertoires derived from bone marrow and spleen of human Ig transgenic mice using a cytokine protein as a model immunogen. We applied a recently established method to immortalize antibody repertoires into yeast display libraries for FACS sorting and in-depth functional screening (57–63), and utilized next-generation sequencing (NGS) and bioinformatic analysis to elucidate the functional and molecular features of antigen-specific (and antigen non-specific) clonal lineages in both bone marrow and spleen antibody repertoires. We identified a collection of anti-cytokine antibodies, some with even double-digit picomolar affinity, and compared the affinity, SHM profile, gene usage, and CDR3 length of antigen-specific antibodies between bone marrow and spleen. Our analysis demonstrates a new ability to analyze multi-organ memory B cell and plasma cell populations to achieve comprehensive profiling of B cell-mediated immunity and for effective therapeutic antibody discovery.

## 2 Materials and methods

### 2.1 Mouse immunization experiment and lymphoid tissues harvest

Trianni human Ig transgenic mice were used as previously described (41, 64). Cohorts of 5 female mice at the age of 8 weeks were immunized with 50 µg of cytokine protein antigen and 50 µl Sigma Adjuvant System (SAS, Sigma-Aldrich, S6322) in 100 µl PBS buffer by intraperitoneal injection five times in 10-day intervals. Sera were collected after the last immunization to assess the antigen-specific antibody titers using the standard ELISA technique. The mice were rested for two weeks and boosted 4 days before harvesting spleen and bone marrow samples for processing. Mice responders 1, 2, and 4 were euthanized, and each mouse’s spleen and femurs were extracted and pooled. Single-cell suspensions of pooled spleen and bone marrow cells were prepared following the depletion of erythrocytes using red blood cell lysis buffer (Sigma-Aldrich). The cells were filtered using a 70 µm cell strainer (BD) and washed twice using 1 × PBS with 5% Fetal Bovine Serum (FBS) by centrifuging at 1500 rpm for 10 mins. Pooled splenocytes and bone marrow cells were resuspended in 1 × PBS with 5% FBS and counted using a Vi-Cell cell counter (Beckman Coulter).

### 2.2 B cell isolation and culture

For bone marrow samples, non-B cells were depleted, and mature murine B cells were isolated from cell suspension using a Pan B Cell Isolation Kit II (Miltenyi Biotec, 130-104-443) *via* Magnetic Activated Cell Sorting (MACS, Miltenyi Biotec, 130-042-301) following the manufacturer’s instructions. Isolated B cells were processed for single-cell paired VH : VL sequencing immediately after selection. 

For spleen samples, mature B cells were enriched from splenocyte cell suspension using the same Pan B Cell Isolation Kit along with Anti-Mouse IgM MicroBeads (Miltenyi Biotec, 130-047-301) for the depletion of naïve B cells. Before proceeding to VH : VL capture, enriched B cells were cultured for five days for an *in vitro* stimulation in Iscove’s Modified Dulbecco’s Medium (IMDM, Gibco, 31980030) supplemented with 10% FBS, 1% MEM Non-Essential Amino Acids, and 1% Penicillin-Streptomycin (Pen-Strep). Cells were co-cultured with 1 × 10^5^/mL irradiated CD40L-secreted 3T3-msCD40L feeder cells (kind gift from John Mascola, VRC, NIAID) together with 100 units/mL IL-2 (PeproTech, 200-02) and 50 ng/mL IL-21 (PeproTech, 200-21) to support B cell expansion and promote antibody gene transcription (65, 66).

### 2.3 Generation of natively paired VH : VL cDNA libraries and antibody yeast display libraries

Immune repertoire sequencing of bone marrow and spleen B cells was performed as previously described (65, 67, 68). Briefly, B cells resuspended in Dulbecco’s phosphate-buffered saline (DPBS) and oligo(dT) mRNA capture magnetic beads prepared in lysis buffer were co-injected into an axisymmetric flow-focusing device together with a rapidly running oil phase surrounding the outside of the needle. Single cells were captured and lysed in emulsion droplets, and B cell mRNAs were annealed to poly(dT) beads. Beads were recovered, washed, and re-emulsified with PCR reaction mix for overlap-extension (OE) reverse transcription PCR (RT-PCR) using SuperScript III One-Step RT-PCR System with Platinum Taq DNA Polymerase (Invitrogen, 12574-026) (**Table S1**). OE RT-PCR joined the VH and VL genes through a linker containing NheI and NcoI restriction sites to form paired VH : VL amplicons. cDNAs were extracted, and a nested PCR using KAPA HiFi DNA Polymerase (Roche, 7958897001) was performed under the following conditions to further amplify and generate the final paired VH : VL amplicon libraries: 2 min initial denaturation at 94°C, denaturation at 94°C for 30 s, annealing at 58°C for 30 s and extension at 72°C for 20 s for 4 cycles, denaturation at 94°C for 30 s, annealing at 62°C for 30 s and extension at 72°C for 20 s for 19 cycles, final extension at 72°C for 7 min. The resulting VH : VL amplicon libraries were prepared for next-generation sequencing *via* 2×300 bp Illumina MiSeq through two-step PCR reactions for adapter and barcode addition. cDNA libraries were also prepared to sequence the VH and VL regions separately to reveal the complete variable region gene sequences.

To construct antibody yeast display libraries, VH : VL cDNA libraries were PCR amplified with primers to include NotI and AscI restriction sites. Resulting PCR products were digested with NotI and AscI and subsequently ligated into yeast display vector pCT-VHVL-K2 double digested with the same enzymes. Ligated products were transformed into *E.coli via* electroporation, and plasmid libraries were maxiprepped. These plasmid libraries were further digested with NheI and NcoI and then ligated with the pre-digested Gal1/Gal10 bidirectional promoter. *E.coli* transformation and maxiprep were performed to obtain the final plasmid DNA libraries (57–59). For yeast transformation, we used a high-efficiency yeast electroporation protocol to transform the library inserts together with pre-digested vector backbone into an electrocompetent AWY101 yeast strain (69). The library inserts were PCR amplified to contain homologous ends to AscI and NotI pre-digested pCT-VHVL-K2 vector, and the final plasmid libraries were completed in yeast *via* homologous recombination. The transformed yeast libraries were passaged twice in SDCAA media (Teknova, 2S0540) to maintain a single copy of plasmid per each yeast cell before being stained for screening. The passaged yeast libraries were referred to as pre-sort libraries.

### 2.4 Antigen production and purification

The cytokine gene was encoded by mono-cistronic plasmids, synthesized, and codon optimized for *Homo sapiens* (GeneArt). The cytokine protein antigen was produced by transient transfection of HEK293 cells using 293Fectin as a transfection reagent, and the transfected cells were cultivated in FreeStyle 293 Expression medium up to 1.5 L. After 7 days of culture, cells were removed by centrifugation, and cell supernatants were filtered through a 0.22 µm membrane. The cytokine antigen with a His tag was purified from clarified supernatants *via* metal affinity chromatography on an ÄKTAxpress instrument using nickel columns. Eluted protein was concentrated *via* centrifugation and quantified by UV spectrometry. The sample was then polished by Size Exclusion Chromatography using Superdex 200 columns, formulated in DPBS buffer, and filtered through a 0.22 µm membrane.

### 2.5 FACS screening of antibody yeast display libraries

Prior to each FACS sorting experiment, each yeast library was inoculated at a final OD_600_ of 0.3 in 10 mL SGDCAA media, SGCAA media (Teknova, 2S0542) supplemented with 2 g/L dextrose and 1% Pen-Strep, for 36 hours at 20°C, 225 rpm to induce Fab expression on yeast surface. Single biotinylated cytokine antigen probes used in the sorting were pre-conjugated to Streptavidin-R-Phycoerythrin (SA-PE, Invitrogen, S21388) at a 3:1 molar ratio (antigen:streptavidin) following fractional addition of SA-PE to cytokine antigen protein to ensure complete saturation of biotin-binding sites. After each round of sorting, sorted yeast cells were cultured and expanded in low pH SDCAA media (pH 4.5) to minimize bacterial contamination for 24-48 hours at 30°C, 225 rpm, and subsequently inoculated in SGDCAA media to prepare for the next round of sorting. 

For the first round of sorting, 3 × 10^7^ yeast cells from each induced pre-sort library were first pelleted down by centrifugation at 1,000 × *g* for 5 mins at 4°C and washed twice with ice-cold staining buffer (1 × PBS with 0.5% BSA and 2 mM EDTA). Subsequently, cells were stained with 100 nM pre-conjugated cytokine antigen and an anti-FLAG-fluorescein isothiocyanate (FITC) conjugated monoclonal antibody (Sigma-Aldrich, F4049) for 30 mins with gentle agitation at 4°C in the dark. Anti-FLAG-FITC was added to detect the FLAG tag next to the antibody light chain and used as a marker for the quantification of Fab VL expression. Cells were washed three times and resuspended in 1 mL cold staining buffer to sort for FITC+PE+ populations (referred to as Round 1 samples). A separate aliquot of 3 × 10^7^ yeast cells from each pre-sort library was stained with anti-FLAG-FITC alone to collect the Fab-expressing populations (referred to as VL+ samples). The second round of sorting was performed following the same protocol, but for only 1 × 10^7^ yeast cells of Round 1 samples resuspended in a final volume of 600 µL staining buffer. The resulting sorted libraries were referred to as Round 2 samples and were subjected to affinity binning of each clone by affinity titration at the third round of sorting. For affinity titration, 5 × 10^6^ yeast cells from Round 2 libraries were stained with anti-FLAG-FITC and pre-conjugated cytokine probes at a final concentration of 0.1 nM, 1 nM, 10 nM, and 100 nM, respectively.

All sorting experiments were performed on a SONY MA900 cell sorter with associated software for gating and analysis. The FACS gating strategy was described previously (57, 61, 70). Flow cytometry data were exported and analyzed by Flowjo (Flowjo 10.4, Flowjo, LLC).

### 2.6 MiSeq preparation, NGS, and bioinformatic analysis

Sorted yeast cells from each round of screening were recovered in low pH SDCAA, and yeast plasmid DNAs were miniprepped as previously described (57, 71). MiSeq preparation for antibody heavy chain genes of each sorted library was conducted through a two-step PCR protocol similar to the one for natively paired VH : VL amplicon libraries described above. Heavy chain genes were first amplified out using primers containing gene-specific and common MiSeq adapter sequences, followed by a second PCR for unique barcode addition to individual libraries using KAPA HiFi DNA Polymerase (Roche, 7958935001). The resulting DNA libraries were sequenced on Illumina 2×300 bp MiSeq platform.

NGS data from paired VH : VL amplicons, VH-only amplicons, and VL-only amplicons were analyzed as previously described (57, 71). Briefly, raw sequences were quality-filtered to remove erroneous reads and subjected to IgBLAST for V-(D)-J gene identification and CDR3 annotation. Sequences with out-of-frame V(D)J recombination were excluded, and the remaining sequences were clustered based on CDR-H3 nucleotide (nt) sequences to 96% nucleotide identity with terminal gaps ignored using USEARCH v5.2.236 (72). For each CDR-H3 cluster, the most abundant CDR-L3 corresponding with it was assigned as its native CDR-L3 pair. The most prevalent CDR-H3:CDR-L3 variant of a cluster was selected as the representative CDR-H3:CDR-L3 pair of that cluster. CDR-H3:CDR-L3 clusters with only one read were removed from further repertoire analysis. The remaining clusters were reported as the final set of unique clusters, which were also used to approximate distinct antibody lineages. Generation of consensus sequences of the complete VH and VL from VH-only and VL-only data corresponding to the dominant representative antibody in each antibody lineage was performed as previously described (58, 68, 73, 74). Framework region (FR) corrections were further conducted on consensus sequences to correct the possible errors introduced by the degenerate bases in OE RT-PCR FR1 and FR4 amplification primers prior to antibody synthesis and expression. A pairing precision analysis between bone marrow and splenocyte samples was carried out as previously described to assess the quality of single-cell sequencing experiments (68, 75).

NGS data of sorted yeast samples were quality-filtered and annotated to identify CDRs and V-, D-, and J-genes as described above and subsequently mapped to the paired VH : VL unique cluster data. Each CDR-H3 sequence was tracked across different sorted samples to determine its functional performance. The frequency (F) of a given CDR-H3 amino acid sequence in a sorted sample was calculated using its reads in this sample divided by the total reads of this sample. The performance of each CDR-H3 in each sorted sample was determined using enrichment ratio (ER), which was calculated as the frequency of a CDR-H3 in the particular sorted sample divided by the frequency of this CDR-H3 in the corresponding VL+ reference sample (58, 61, 70).

### 2.7 mAb production

IgGs were generated using Linear Expression Cassettes (LEC) as previously described (64). Briefly, antibody heavy and kappa variable region gene fragments were synthesized (GeneArt) including 5’ overhang sequences overlapping a CMV promoter gene segment and 3’ overhang sequences complementary to the N-terminus of respective human heavy or kappa constant region gene fragments. The three separate gene fragments were assembled into LECs by overlap-extension PCR using KOD DNA polymerase (Merck). 0.6 µg heavy chain LECs and 0.6 µg light chain LECs for each antibody were mixed directly in 96-well PCR plates for co-transfection.

Expi293F cells (Gibco, A14527) were cultured in serum-free Expi293 Expression Medium (ThermoFisher, A1435101) in a 37°C orbital shaker with 8% CO_2_ and 80% humidity at 150-200 rpm. On the day before transfection, cells with a viability of at least 95% and a density less than 5 × 10^6^ cells/mL were diluted, resuspended, and cultured in 80 mL fresh pre-warmed Expi293 Expression Medium at 2.7 × 10^6^ cells/mL until the day after. Transfection was performed in 2-mL 96 deep well blocks (Greiner, 780271) in a final volume of 1 mL. A working cell suspension was prepared with a final density of 3 × 10^6^ cells/mL. Cells were plated in 96 deep well blocks in a final volume of 735 µL/well and incubated for one hour in Multitron orbital shaker with 3 mm orbitals at 1,000 rpm. For each 1-mL transfection, lipid-DNA complexes were prepared in 96-well polypropylene plates (Greiner, 650261) using Hamilton Star Plus automated. Briefly, 1μg of linear DNA (LEC) and 3.8 μL of ExpiFectamine 293 Reagents (Gibco, A14524) were first diluted in Opti-MEM I Reduced Serum Medium to a total volume of 105 µL and incubated for 5 minutes at room temperature (RT), respectively. After the incubation, diluted linear DNA and diluted ExpiFectamine 293 Reagents were mixed to obtain a total volume of 210 µL and incubated for 20 minutes at RT. 210 µL of mix complexes were added to each well of the suspension cell plate, and after 20-24 hours of incubation in the 37°C shaker, 5 µl of ExpiFectamine 293 Transfection Enhancer 1 and 50 µl of Enhancer 2 were added to each well to reach a final volume of 1 mL in each well of the 96-well plate. The transfected cells were incubated for another 5 days and were harvested by centrifugation at 3000 × g for 10 mins. The supernatants were then removed and filtered through 0.22 μm 96-well filter plates (Agilent) and collected in sterile 2 mL 96 deep well blocks.

To assess antibody expression, 40 µL of the cell media containing transfected cells were collected, sterile filtered, and plated into Octet 384 TW-well plates (Sartorius, 18-5080). The supernatants were analyzed using the Octet HTX system (Sartorius Octet, RH96) with a Protein A biosensor-based quantitation assay to quantify human IgG in solution. The average concentration calculated for this campaign was 82 ± 25 µg/mL. The supernatants were normalized to a concentration of 20 µg/mL using the Janus G3 automated liquid handling workstation (PerkinElmer).

### 2.8 SPR binding experiment

SPR-based kinetics experiments were performed on a Carterra LSA instrument equipped with an HC200M chip (Carterra, 4287) at 25°C in an anti-human Fc capture setup using 1x HBS-EP+ buffer (10 mM HEPES, pH 7.4, 150 mM NaCl, 3 mM EDTA, 0.05% v/v Surfactant P20, Cytiva, BR100669) as running buffer. After a priming step with 10 mM MES buffer pH 5.5 (Alfa Aesar, J63341), the carboxyl groups on the chip were activated with a freshly prepared mixture of 1:1:1 v/v/v 400 mM M EDC (ForteBIO, 25952-53-8) + 200 mM sulfo-NHS (ForteBio, 106627-54-7) + 10 mM MES buffer (pH 5.5). In the coupling step, goat anti-human IgG Fc antibody (SouthernBiotech, 2048-01) at a concentration of 75 µg/mL diluted in 10 mM sodium acetate buffer (pH 4.5, Bruker, SAB1045-250) was flowed over the activated surface. In the blocking step, excess activated carboxyl groups were blocked with 1 M ethanolamine (pH 8.5, Bruker, 1862666), yielding final coupled anti-human IgG Fc antibody levels of 11873 ± 226 (mean ± standard deviation) Response Units (RU). Next, the immobilized chip was primed with running buffer followed by conditioning with 10 mM Glycine (pH 2.0, Bruker, 1862654). Crude antibody extracts at a concentration of either 2 µg/mL or 4 µg/mL diluted in running buffer were printed with the 96-print head on the discrete anti-human Fc antibody spots, yielding a ligand surface with capture levels ranging from 100 to 3600 RU. Anti-human Fc surface spots without crude extracts were also included as blank surfaces for referencing purposes. A concentration series of cytokine protein with nine increasing concentrations ranging from 150 pM to 1 μM (3-fold dilution series prepared in running buffer) were injected sequentially as analytes to the antibody-anti-human Fc spots and blank surface spots for 300 seconds followed by a dissociation phase of 1800 seconds. Data processing was done using Carterra’s Kinetics Software (Version 1.7.1.3055). Data were double referenced by subtraction of a reference ligand lane (analyte sampled over the blank surface) and a blank buffer injection (running buffer sampled over the ligand surface). To extract the kinetic parameters, the data were analyzed in the framework of the Langmuir 1:1 binding model. The lower quantification limit of the off-rate was calculated based on the widely accepted 5% decrease in the binding signal (76). If the fitted off-rate was below the quantification limit, the lower quantification limit was reported and used for K_D_ determination. For the calculation of the lower quantification limit of each interaction, the following formula with the RU of the highest concentration (1 µM) and the dissociation time (t_dissociation_) was used:


$$[\ln\frac{Min\left(\left(RU_{1\text{µM}}*95\%\right),\left(RU_{1\text{µM}}-5\right)\right)}{RU_{1\text{µM}}}]/\left(-t_{dissociation\;}\right)$$

### 2.9 Statistical analysis

Statistical significance for the level of somatic hypermutation and CDR-H3 amino acid length was determined using the Kolmogorov-Smirnov test with a Bonferroni correction used to correct for multiple comparisons. Statistical significance for K_D_, k_on_, and k_off_ was determined using the one-way ANOVA (Kruskal-Wallis) test or the Mann-Whitney U test. A Pearson correlation was used to measure the strength of the relationship between Round 2 ER and observed affinity (K_D_). All statistical and correlation analyses were conducted using GraphPad Prism 9 software.

### 2.10 Replication of experiments

This experimental process can be repeated with any human cytokine antigen for immunization and library screening. Similar results would be expected by following the detailed methods using the same materials as specified above.

## 3 Results

### 3.1 High-throughput sequencing of natively paired VH : VL repertoires

**Figure 1:**
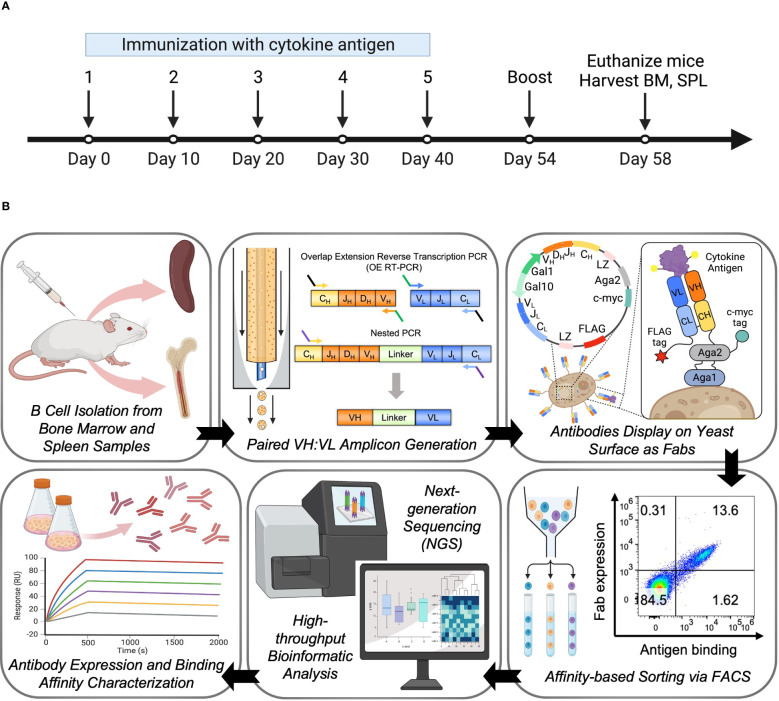
Large-scale antibody response characterization and effective antibody discovery from the bone marrow and spleen compartments of immunized mice. **(A)** Overview of mouse immunization with cytokine antigen. Human Ig transgenic mice (n=5) were immunized with the cytokine antigen and boosted before being euthanized for spleen and bone marrow tissue isolations. **(B)** Experimental overview. Bone marrow and spleen were harvested from mice immunized with cytokine antigen, and mature B cells were isolated from pooled single-cell suspensions. B cells were subjected to a flow-focusing device where a single B cell was captured and lysed in a single emulsion droplet, and its mRNAs were annealed to the poly(dT) beads. OE RT-PCR and nested PCR were used to isolate natively paired VH : VL amplicons. Paired VH : VL amplicons were cloned into yeast display vectors to construct antibody yeast display libraries. Yeast display libraries were screened against cytokine antigens by fluorescent-activated cell sorting (FACS). Sorted libraries were submitted for next-generation sequencing (NGS) of antibody genes, and bioinformatic analysis was performed to determine the binding affinity characteristics and genetic features of the clones in the libraries. Hits were selected for full IgG expression and *in vitro* binding characterization.

Five humanized transgenic mice were immunized intraperitoneally with cytokine antigens, and the serum antibody titers of each mouse were measured. All mice generated measurable serum titers against the immunization, and responders ranked 1, 2, and 4 were sacrificed 4 days after the last booster injection. Spleen and both femurs of each mouse were extracted, and single-cell suspensions of 3 mice were pooled and counted (**Figure 1A**). For B cell selection, around 56% and 78% of the bone marrow cells and splenocytes were isolated as mature B cells or IgM-negative mature B cells using magnetic cell sorting, respectively. In total, 4 million bone marrow B cells and 3.35 million spleen B cells were emulsified for natively paired VH : VL amplicon libraries generation as previously described (65, 67) (**Figure 1B** and **Table S2**). We specifically focused on IgG repertoires, as most of the highest affinity antibodies elicited by intraperitoneal immunization belong to the IgG isotype. The transgenic mouse strain used in this study was humanized on the heavy and kappa V(D)J segments, thus we used primers that target only IgG heavy chain and IgK light chain isotypes to specifically amplify IgG/IgK antibody repertoires for library generation and downstream analysis (**Table S1**). Paired VH : VL amplicons showed the expected 850 bp approximate size and were sequenced *via* the Illumina MiSeq platform.

NGS data analysis was performed using our established bioinformatic pipeline, which was designed to mitigate NGS errors in antibody repertoire sequencing by employing multiple quality filters across different processing steps (73). An antibody cluster is a bioinformatic approximation of a unique antibody lineage, and we used the number of unique clusters as a rough estimation of genetic diversity in terms of B cell lineage in these two libraries. For bone marrow- and spleen-derived IgG libraries, we identified 3,204 and 1,121 unique VH : VL antibody clusters, respectively (**Table S2**). Many clusters contained somatic variants with ≥96% CDR-H3 sequence identity, and the most prevalent sequence of each cluster was selected as the representative clone for that unique cluster. These representatives and their closely related somatic variants accounted for more than 80% of the total annotated VH : VL reads in the quality-filtered library (**Table S2**), suggesting that the IgG repertoires of these mice were heavily polarized with a small fraction of the clones expanding excessively *in vivo* to occupy the majority of reads in these samples as a result of the repetitive immunizations with cytokine antigen. Furthermore, the exclusion of single-read CDR-H3:CDR-L3 antibody sequences from the data eliminated a large fraction of low-frequency antibodies from large B cell populations.

### 3.2 Generation and FACS-based screening of antibody yeast display libraries

**Figure 2:**
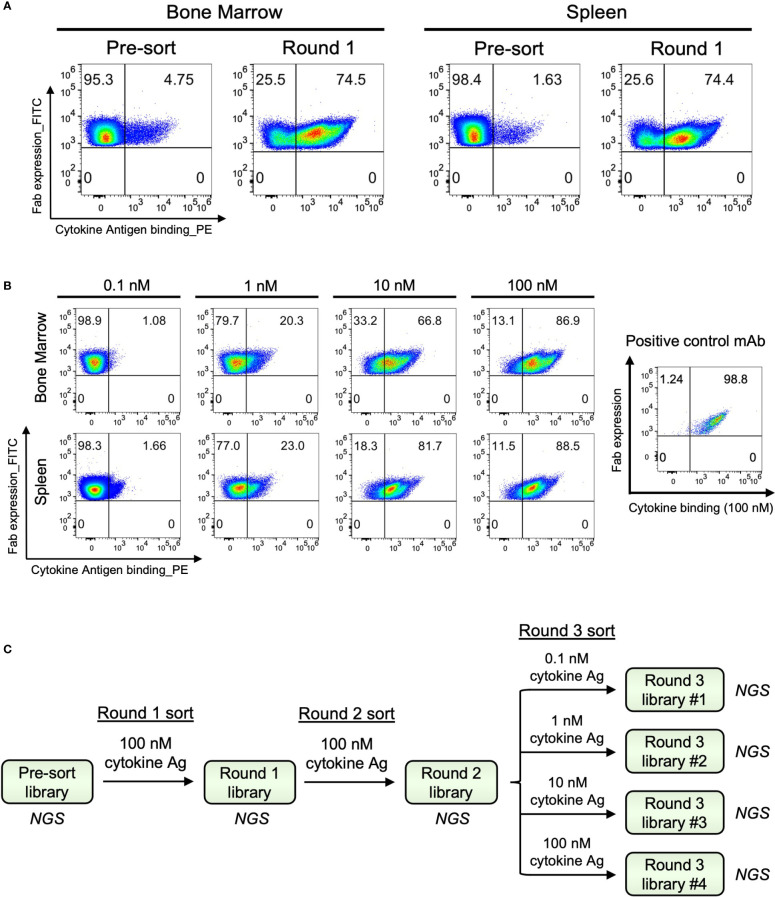
Functional screening of bone marrow- and spleen-derived antibody libraries from immunized mice. **(A)** Representative FACS plots for one round of antibody yeast library enrichment screening against cytokine antigen. Surface Fab expression was detected by an anti-FLAG-FITC mAb, and antigen binding was detected by PE-conjugated antigen. **(B)** Affinity titrations were performed on the enriched libraries using an antigen concentration series to bin each clone in the libraries by their individual antigen-binding affinity. A positive control mAb with known binding affinity against the cytokine was included for comparison (K_D_=1.3E-07 M, k_off_=1.4E-02 s^-1^, k_on_=1.1E+05 M^-1^s^-1^). **(C)** FACS-based enrichment sorting and affinity titration overview.

Natively paired VH : VL amplicon libraries were cloned into a yeast surface display vector to generate antibody display libraries to screen for antigen binding *via* FACS. (**Figure 1B** and **Figure S1**). Each cloning step was completed with at least 7 × 10^5^ transformants to ensure sufficient coverage of VH : VL amplicon libraries (**Table S3**). We analyzed the bone marrow- and spleen-derived IgG libraries with fluorescently labeled cytokine antigens by FACS (**Figure S2**). Among the Fab-expressing populations, approximately 4.8% of BM-derived yeast cells and 1.6% of SPL-derived yeast cells in the pre-sort libraries bound to the cytokine antigen. After only one round of enrichment, the antigen-binding population increased to around 74% for both libraries, demonstrating highly effective enrichment of antigen-specific clones that further supported sequence-based observations of high polarization for antigen-specific antibodies in these repertoires (**Figure 2A**). The antibody responses of these two libraries against the cytokine antigen were substantially higher than other antibody screening studies conducted by our group that utilized human peripheral memory B as the source for cell repertoires (57–61). We further binned the yeast display repertoires into high-, medium-, and low-affinity populations for binding to cytokine antigen using affinity titrations that consisted of sorting enriched libraries against a concentration series of the cytokine (0.1, 1, 10, and 100 nM, **Figure 2B**) (57, 58, 70, 77). All sorted samples were sent for NGS and subsequent bioinformatic mining of functional repertoire data (**Figure 2C**).

### 3.3 NGS-based repertoire-scale functional analyses

**Figure 3:**
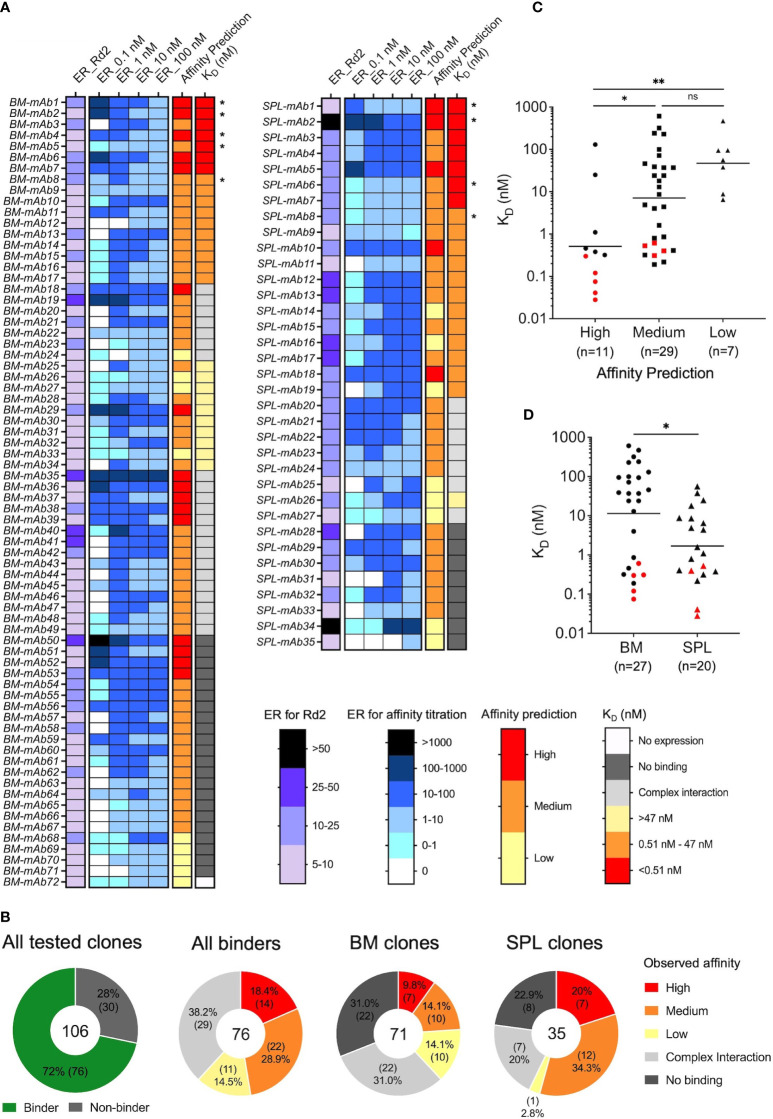
Comprehensive affinity characterization of antigen-specific clones from bone marrow and spleen antibody repertoires. **(A)** Heatmap of functional analysis results for bone marrow and spleen antibody libraries, including bioinformatic calculated enrichment ratio (ER) after two rounds of screening, ER for affinity titrations, predicted affinity based on yeast screening data, and the observed affinity (K_D_) measured by SPR. Some clones showed complex interaction profiles in SPR data, and their K_D_ were considered indicative. Clones were ranked by K_D_ from smallest to largest. Clones that showed no binding and complex interaction were ranked by predicted affinity from high to low. Clones marked with an asterisk (*) had k_off_ below the quantification limit, and their k_off_ were calculated based on the response level of the highest concentration and the dissociation time. BM: bone marrow. SPL: splenocyte. **(B)** Composition of clones with SPR-confirmed no-, high-, medium-, and low-affinity antigen binding in different analysis groups. **(C)** Correlation between predicted affinity and observed affinity for antibody binders; antibodies with complex interactions by SPR were omitted. Each point represents a single antibody clone. Red points represent the clones that had k_off_ below the quantification limit. Bar represents the geometric mean value of the group. A one-way ANOVA (Kruskal-Wallis) test was used to determine statistical significance (**p< 0.01, *p< 0.05). **(D)** Comparison of the observed affinity between bone marrow and spleen antibody binders; antibodies with complex interactions by SPR were omitted. Red points represent the clones that had k_off_ below the quantification limit. Bar represents the geometric mean value of the group. Statistical significance was determined by the Mann-Whitney U test with *p< 0.05.

We conducted large-scale computational mining of yeast sort NGS data to reveal the antigen binding features of each antibody clone, including both antigen specificity and coarse-grain affinity. Lineages were first collapsed into clonal representatives using CDR-H3 clustering. To determine the antigen specificity of each CDR-H3 clonal representative, we calculated the enrichment ratio (ER) of each clone across screening rounds. Clones with more than 2 reads in the VL+ library and an ER larger than 5 at Round 2 were considered antigen-specific. In total, we identified 72 and 35 antibody clones from bone marrow and splenocyte libraries from yeast sorting data, respectively. These 107 clones were selected for full IgG synthesis and *in vitro* characterization (**Figure 3A**). We also predicted the coarse-grain affinity for each of these clones by comparing the ER of a given clone across the four antigen concentration sorts. We considered the clones with the highest ER in the 0.1 nM cytokine antigen sorted library as high-affinity binding clones, those with the highest ER in the 1 nM or 10 nM sorts as medium-affinity binding clones, and the ones with the highest ER in the 100 nM sort as low-affinity binding clones (**Figure 3A**).

### 3.4 *In vitro* binding and affinity characterization

We evaluated the binding affinity of the 107 selected clones against cytokine protein by SPR, revealing that 76 of the 107 antibodies (72%) were genuine binders, 30/107 antibodies (28%) showed no binding, and one antibody did not express as full IgG (**Figures 3A, B** and **Tables S4**, **S5**). These data revealed that the hit selection based on Round 2 ER>5 and VL+ >2 reads was efficient but not perfect. The hit rate was slightly lower compared to our previous studies using human B cell repertoires (72% vs. 90% - 100%), likely due to the larger size of the selected pool for characterization here (~100 vs. ~20 clones), and potentially the differences in B cell tissue origins, cell selection strategies, and sort designs (58, 60, 61). The genuine binding clones from BM and SPL comprised about 5-6% of reads in the BM or SPL clustered populations, further confirming the polarized nature of these antibody libraries (**Tables S4**, **S5**).

For some antibodies, we failed to obtain an accurate K_D_ value generally due to one of two reasons: 1) 9 clones had a k_off_ so low that it was below the quantification limit for accurate calculations, and 2) 29 clones showed complex interaction profiles in SPR measurement and their data could not be properly fit by the software using a 1:1 Langmuir fit (**Figures S3A, B**). Clones in both situations were effectively validated as antigen binders, and the clones with complex interaction profiles were excluded from the main figure affinity analyses and comparisons. (The entire sets of affinity assignments are presented in **Figure S4** for further reference.) We considered the kinetic parameters of binders with k_off_ below the quantification limit as exploitable and included these clones in all analyses (**Figure S4A**). k_off_ values were overestimated for these antibodies because the experimental dissociation phase was not long enough to quantify the dissociation constant between mAbs and antigen accurately. Therefore, such clones bind the antigen even tighter than the K_D_ value suggests.

We compared the range and geometric means of K_D_ values of each predicted affinity group. While the K_D_ distribution of the predicted medium-affinity group was not statistically different from the K_D_ of the predicted low-affinity group due to overlapping variability between groups, the majority of clones in the predicted high-affinity bin displayed subnanomolar K_D_ values, and the K_D_ geometric means were different between predicted high- and medium-affinity bins, and between predicted high- and low-affinity bins (**Figures 3C**, **S4B**). As expected based on our previous studies (57, 58, 60, 61), the magnitude of Round 2 ER did not correlate with observed affinity (**Figure S4C**). We further examined the correlation between the k_on_ or k_off_ of each clone with its predicted affinity, and we found that neither of them correlated with the predicted affinity better than K_D_ (**Figure S4D**), suggesting that binding events assayed on the yeast surface between Fabs and antigens were influenced by both binding on-rates and off-rates (**Figure S4E**). In addition, the majority of clones in the predicted high-affinity bin had higher affinity k_on_ and lower k_off_ values compared to the rest of the clones, demonstrating the efficient affinity prediction for high-affinity binders.

We assigned thresholds of K_D_ value for observed affinity bins based on the geometric means of K_D_ for the predicted high- (0.5 nM) and low-affinity (47 nM) groups. In total, we identified 14 high-affinity binders with K_D_ below 0.5 nM (7 from BM and 7 from SPL), 22 medium-affinity binders with K_D_ ranging between 0.5 nM and 47 nM (10 from BM, 12 from SPL), and 11 low-affinity binders with K_D_ higher than 47 nM (11 from BM and 1 from SPL (**Figure 3B**). The yeast affinity titration allowed correct categorization for about 60% of the clones (29/47), and the classification of high- and medium-affinity clones was more precise than for low-affinity mAbs (**Figures 3A, B**). Antibodies originating from the spleen displayed statistically higher antigen binding affinity compared to mAbs from the bone marrow (**Figures 3D**, **S4F**). The statistically different affinities for clones across BM and SPL groups were consistent with the earlier observation in our yeast screening data that the spleen library showed saturated binding signal at 10 nM of cytokine antigen, while the bone marrow library reached saturation at 100 nM (**Figure 2B**).

### 3.5 Molecular comparisons of bone marrow- and spleen-derived antibody repertoires

**Figure 4:**
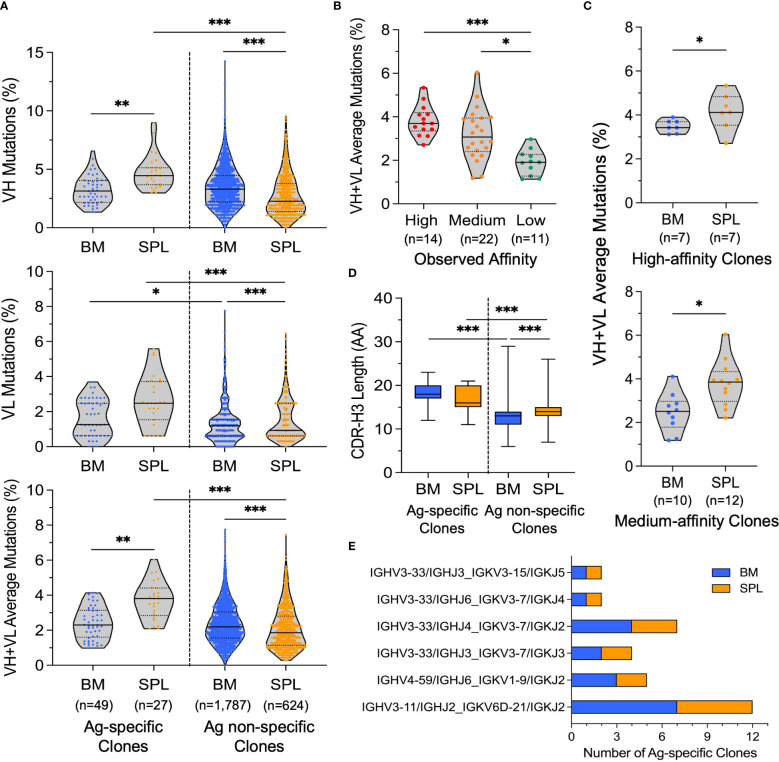
Genetic comparisons of antibody sequences between bone marrow- and spleen-derived antibody libraries. **(A)** Percentage of SHM in the heavy chain variable region (upper), light chain variable region (middle), and both VH and VL together (lower). All SHM analyses reported SHM levels in antigen (Ag)-specific clones (SPR-confirmed binding clones) and antigen (Ag) non-specific clones. Kolmogorov-Smirnov tests were conducted to determine statistical significance (***p< 0.00025, **p< 0.0025, *p< 0.0125 after Bonferroni correction was used to correct for multiple comparisons). All statistically significant comparisons were noted, and non-statistically significant comparisons were omitted. **(B)** Average VH : VL SHM percentage of SPR-confirmed high-, medium-, and low-affinity clones (clones with complex interactions by SPR were excluded). Pairwise K-S tests were used for statistical analyses (***p< 0.0003, **p< 0.0033, *p< 0.0167 after Bonferroni correction). All statistically significant comparisons were noted, and non-statistically significant comparisons were omitted. **(C)** Comparison of average VH : VL SHM percentage in SPR-confirmed high- (upper) and medium- (lower) affinity clones (clones with complex interactions by SPR were excluded) from bone marrow and spleen libraries (K-S test, *p< 0.05). **(D)** Comparison of CDR-H3 amino acid length for antigen-specific clones (SPR-confirmed) and antigen non-specific clones of bone marrow and spleen libraries. BM Ag-specific: 17.9 ± 2.25 (n=49), SPL Ag-specific: 16.9 ± 2.72 (n=27), BM Ag non-specific: 13.1 ± 2.56 (n=3,153), SPL Ag non-specific: 14.1 ± 2.76 (n=1,091) (mean ± standard deviation). Pairwise K-S tests were used for statistical analysis (***p< 0.00025, **p< 0.0025, *p< 0.0125 after Bonferroni correction). All statistically significant comparisons were noted, and non-statistically significant comparisons were omitted. **(E)** The number of SPR-confirmed antigen-specific antibody clones from bone marrow and spleen libraries that shared the same IGHV/IGHJ : IGKV/IGKJ gene usage and also had the same CDR3 amino acid length.

We next sought to understand if there were discrepancies between the genetic features of BM and SPL repertoires and to better understand potential drivers of the affinity differences observed across groups. We first determined the somatic hypermutation (SHM) level of the 76 SPR-confirmed antigen-specific clones and antigen non-specific clones of both bone marrow and spleen. SHM analyses were conducted on the consensus sequences of each unique cluster representative. The rest of the unique cluster representative clones excluding the SPR-confirmed binding clones were considered as antigen non-specific clones. Overall, we observed that antigen non-specific clones from bone marrow showed slightly higher SHM levels than antigen non-specific spleen libraries, and the differences between distributions were statistically significant for all three comparisons tested (VH gene, VL gene, and VH+VL gene) (**Figure 4A**). In contrast, the spleen antigen-specific clones showed consistently higher SHM compared to bone marrow antigen-specific clones, and these differences were statistically significant for the VH gene and VH+VL genes, but the differences were not significant for the VL gene (**Figure 4A**). Antigen-specific clones from the spleen showed higher SHM than spleen antigen non-specific clones; however, the SHM levels for antigen-specific clones in the bone marrow library closely matched the SHM levels for bone marrow antigen non-specific clones (**Figure 4A**). We focused our SHM analyses in **Figure 4A** on comparing antigen-specific clones with antigen non-specific clones (rather than including the entire repertoire as a comparison group) because a substantial fraction of antibody sequences in the entire repertoires comprised antigen-specific clones (5-6%). We also compared the SHM levels between SPR-confirmed binding affinity groups and found that high- and medium-affinity antibodies showed higher SHM compared to low-affinity mAbs (**Figure 4B**). In addition, the high- and medium-affinity antibodies from the spleen showed higher SHM than those from the bone marrow (**Figure 4C**). These data suggested that, in general, BM PC antibodies have higher SHM than SPL antibodies in these mice, but that in our experiment, the ongoing germinal center reactions elicited by repeated immunization enabled the SHM levels of SPL antigen-specific antibodies to exceed that of BM PC antigen-specific clones.

We also investigated the gene usage and CDR3 amino acid lengths of antibodies contained in the bone marrow and spleen libraries. Average CDR-H3 amino acid (AA) lengths of bone marrow and spleen SPR-confirmed antigen-specific clones were similar to each other, but the average CDR-H3 AA length of antigen-specific clones was higher than the rest of the repertoires, and these differences were statistically significant (**Figures 4D**, **S5A**). The antigen non-specific spleen repertoire had a slightly longer CDR-H3 length compared to the antigen non-specific bone marrow repertoire, and this difference was statistically significant (**Figure 4D**). When expanding the sequence analysis from antigen-specific clones to explore the immunogenetics of antigen-specific lineages, we observed that a large fraction of antigen-specific lineages from both libraries were encoded by the IGHV3-33, IGHV3-11 and IGHV4-59 heavy chain V genes, and by the IGKV1-9, IGKV3-7 and IGKV6D-21 kappa chain V genes (**Figure S5B**). We also investigated clonal expansion events in SPR-confirmed antigen-specific clones of bone marrow and spleen libraries. We found six expanded antigen-specific lineages that contained lineage members in both SPL and BM libraries (**Figure 4E**). These clones shared the same IGHV/IGKJ : IGKV/IGKJ usage, had matched CDR-H3 length, and showed high homology in non-templated bases (particularly in the non-templated CDR-H3 and CDR-L3), yet still each clone encoded their own unique mutations (e.g., **Figure S6**), which indicated that these clones were expanded clonal variants that derived from the same V(D)J recombination event and underwent further somatic hypermutation in the course of the immune response.

## 4 Discussion

The rapid identification of high-affinity binding antibodies is greatly desired for antibody discovery campaigns because they can reduce the need for laborious downstream antibody engineering efforts to improve affinity and binding properties. This is especially true for cytokine antagonist mAbs, which are commonly used in inflammation and cancer immunotherapy. To efficiently neutralize cytokines like TNFα, IL-2, IL-4, IL-6, IL-10, IL-12, IL-13, IL-23, IL-33, and others, mAbs need to reach pM affinity for their targets because the cytokine proteins are soluble (78, 79). In this study, we leveraged several techniques for high-affinity human antibody discovery including the use of a transgenic mouse strain, high-throughput paired antibody heavy and light chain sequencing, yeast surface display, and next-generation sequencing to functionally analyze antibody repertoires from bone marrow and spleen tissues *en masse.* In particular, traditional antibody discovery technologies are unable to analyze the functional features of antibodies encoded by plasma cells; our study thus reports a valuable comparison of antibody genotype and phenotype in spleen B cells and bone marrow plasma cells in an immunization model. We revealed a panel of anti-cytokine antibodies and studied their genetic and functional features across lymphoid organs, including genetic comparisons to other cytokine non-specific antibodies from immunized mice.

While our input cells for BM repertoire analysis consisted of all B cells to minimize the loss of precious BM PCs, we consider the BM antibody responses in this study as mainly IgG+ BM PCs because of two reasons. First, PCs constitute the majority of BM B cells, and naïve B cells and immature and transitional B cells do not express IgG. Memory B cells only comprise a small portion (around 9%) of the BM B cell populations, and an even smaller fraction (~15%) of those express IgG transcripts that were analyzed here. Thus, the vast majority of IgG-expressing BM cells were BM PCs (80). Second, PCs express much higher levels of immunoglobulin mRNA transcripts than memory B cells (38), and the PC-derived transcripts amplify much more quickly in RT-PCR. Thus, the PC-derived amplicons appear at earlier PCR cycles and dominate the RT-PCR products. Based on these factors, our study conducted overwhelmingly IgG+ PC analysis in BM, even though we did not specifically purify the IgG+ PC population from BM samples.

The anti-cytokine antibodies that we identified had K_D_ ranging between 28 pM to 610 nM, with the highest affinity antibodies displaying similar or better affinity compared to previously published benchmark fully human anti-cytokine antibodies like Guselkumab (anti-IL-23 mAb, K_D_ = 8 - 62 pM) (81), Secukinumab (anti-IL-17A mAb, K_D_ = 44 - 76 pM) (82), and Adalimumab (anti-TNFα mAb, K_D_ = 99 - 154 pM) (83). Our study focused on the use of a cytokine as a model immunogen to compare genotypic and phenotypic responses across different lymphoid organs, and thus we did not examine additional functional features of the selected mAbs and the *in vivo* efficacy in animal models. We conducted affinity titrations with multiple antigen concentrations (77, 84) rather than simpler affinity gating as in some previous studies (57, 58, 70, 85) to more accurately predict the relative binding affinity of antibodies in our yeast surface display platform. We used affinity titrations because the different titration groups can analyze a broader range of antibody affinities than affinity gating, which uses only a single (albeit carefully selected) antigen concentration. In this study, we synthesized and expressed only the most prevalent representative antibody sequence of each unique CDR-H3:CDR-L3 cluster. We note that somatic variants were present for many of the sequence clusters, and these somatic variants can also be mined from sequencing and functional datasets for additional antibody discovery and optimization efforts.

Our comparison of functional and genetic features between bone marrow and spleen antibody repertoires revealed that antibodies isolated from the spleen had a higher average affinity compared to antibodies isolated from the bone marrow (**Figure 3D**), which may be explained by the higher somatic hypermutation level exhibited in spleen antigen-specific clones compared to bone marrow antigen-specific clones (**Figure 4A**). Because we were analyzing the entire B cell populations in the spleen and bone marrow, we suspect that this difference may be driven by GC B cells in the spleen that had gone through more cycles of affinity maturation and class-switched to IgG, but had not yet differentiated into plasma cells and relocated to bone marrow at the time when tissue samples were collected; it has been reported that it takes around six days for enriched antigen-specific bone marrow plasma cells to appear (86). There is also a possibility that some GC B cells may have obtained some additional mutations during the *in vitro* stimulation with CD40L and IL-21, but this is less likely as a previous study showed no detectable VH gene mutations after 8-day stimulation with IL-21 of the *in vitro* induced GC B cells (87). We also noticed that, in general, higher-affinity antibodies maintained a higher level of SHM compared to lower-affinity antibodies. This observed correlation between affinity and SHM level was consistent with the widely accepted notion that the higher developed SHM during affinity maturation usually leads to better affinity and potency of mAbs in simple immunization models (88, 89). We also found multiple antigen-specific clones from these two libraries that were clonal variants, documenting a level of clonal expansion across tissues during B cell maturation for the antigen-specific cell groups. We also noted that within each clonal cluster, some variants displayed different binding affinities than others. Further structural analyses may be conducted to fully understand the influence of unique mutations within clonal lineages on antigen binding and antibody performance.

In summary, we demonstrated that our platform could effectively analyze both splenic B cell and plasma cell repertoires and identify antigen-specific lineages along with coarse-grain relative affinity predictions. Our platform also provides a valuable tool for in-depth analysis of functional and genetic features of antibody repertoires across spleen and bone marrow compartments to enhance our fundamental understanding of antibody immunity.

## Data availability statement

The data generated for this study are deposited in the NCBI repository, accession number PRJNA909274. Due to legal requirements and confidentiality agreements, the specific cytokine antigen used in this study is undisclosed. Additional experimental information can be shared upon request by contacting the corresponding authors and after the completion of the appropriate MTA/CDA documents.

## Ethics statement

All experiments were approved and conducted in AAALAC (The Association for Assessment and Accreditation of Laboratory Animal Care International) approved animal facility in accordance with the guidelines of Sanofi Genzyme Institutional Animal Care and Use Committee.

## Author contributions

XP, SLA, CAB, JMD, EV, MAS, and BJD designed the experiments. XP, SLA, CC, EDT, PSL, SPR, DA-R, MR, and MOS performed the experiments. XP, EDT, AF, DA-R, MG-G, and BJD analyzed the data. XP, MAS, and BJD wrote the manuscript with feedback from all authors. All authors contributed to the article and approved the submitted version.

## Supplementary material

Supplementary Figure 1 VH : VL library cloning overview into the yeast display vector. Paired VH : VL amplicons were cloned into yeast display vector using an initial PCR amplification step, followed by a two-step restriction digestion and ligation reactions.

Supplementary Figure 2 Representative FACS gating strategy for yeast display functional screening. Yeast cells were stained with anti-FLAG_FITC and cytokine_PE for sorting.

Supplementary Figure 3 Representative SPR sensorgrams for antibody binding to cytokine protein. **(A)** Representative mAbs from the bone marrow IgG library. **(B)** Representative mAbs from the spleen IgG library.

Supplementary Figure 4 Predicted affinity vs. observed affinity analyses. **(A)** A scatter plot of k_on_ vs. k_off_ for genuine antibody binders; antibodies with complex interactions by SPR were omitted, and points were categorized by observed affinity. Red points represent the clones that had k_off_ below the quantification limit. **(B)** Correlation between predicted affinity and observed affinity for all binding antibodies tested. Each point represents a single binder. Red points represent the clones that had k_off_ below the quantification limit. Blue points represent the clones that had complex interactions, which were omitted from **Figure 3C**. Bar represents the geometric mean value of the group. No statistical significance was observed across each affinity group by a one-way ANOVA (Kruskal-Wallis) test. **(C)** Correlation between Round 2 ER and observed affinity (K_D_) for genuine antibody binders; antibodies with complex interactions by SPR were omitted. Pearson correlation coefficient r and *p* value are shown in the plot. **(D)** Correlation between predicted affinity and observed k_on_ (left) and k_off_ (right) for genuine antibody binders; antibodies with complex interactions by SPR were omitted. Red points represent the clones that had k_off_ below the quantification limit. Bar represents the geometric mean value of the group. A one-way ANOVA (Kruskal-Wallis) test was used to determine statistical significance (***p*< 0.01, **p*< 0.05). **(E)** A scatter plot of k_on_ and k_off_ for genuine antibody binders; antibodies with complex interactions by SPR were omitted, and points were categorized by predicted affinity. Red points represent the clones that had k_off_ below the quantification limit. **(F)** Comparison of observed affinity between all bone marrow and spleen binding antibodies. Red points represent the clones that had k_off_ below the quantification limit. Blue points represent the clones that had complex interactions, which were omitted from **Figure 3D**. Bar represents the geometric mean value of the group. Statistical significance was determined by the Mann-Whitney U test.

Supplementary Figure 5 Genetic analysis of bone marrow and spleen antibody repertoires. **(A)** CDR-H3 (upper) and CDR-L3 (lower) amino acid length distributions for the SPR-confirmed antigen-specific clones (right) and the rest of the repertoires (left) of bone marrow and spleen libraries. **(B)** IGHV/IGHJ and IGKV/IGKJ gene usage for the SPR-confirmed antigen-specific lineages and overall antibody repertoires of bone marrow and spleen libraries.

Supplementary Figure 6 Representative multiple sequence alignments of antibodies from bone marrow and spleen libraries that shared the same gene usage of IGHV4-59/IGHJ6*02_IGKV1-9/IGKJ2*01. Alignments were shown for both heavy chain and light chain amino acid sequences and nucleotide sequences.
