# Supplementary figures and images for: Large-scale antibody immune response mapping of splenic B cells and bone marrow plasma cells in a transgenic mouse model
Source: Front Immunol. 2023 Jun 5;14:1137069. doi: 10.3389/fimmu.2023.1137069 (PMC10280637; doi:10.3389/fimmu.2023.1137069)

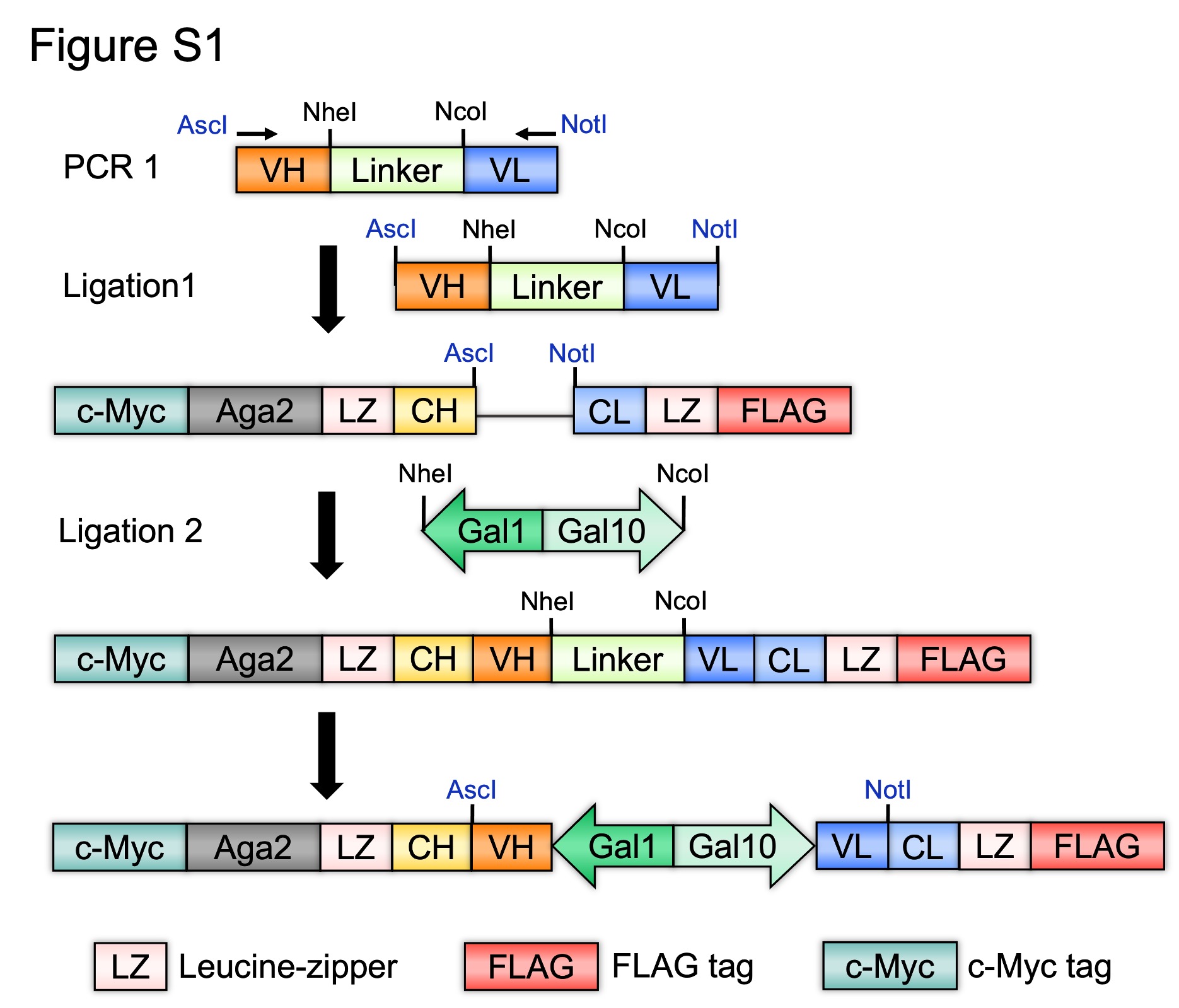

Supplement: Supplementary Figure 1 — VH : VL library cloning overview into the yeast display vector. Paired VH : VL amplicons were cloned into yeast display vector using an initial PCR amplification step, followed by a two-step restriction digestion and ligation reactions. [file Image_1.jpg]

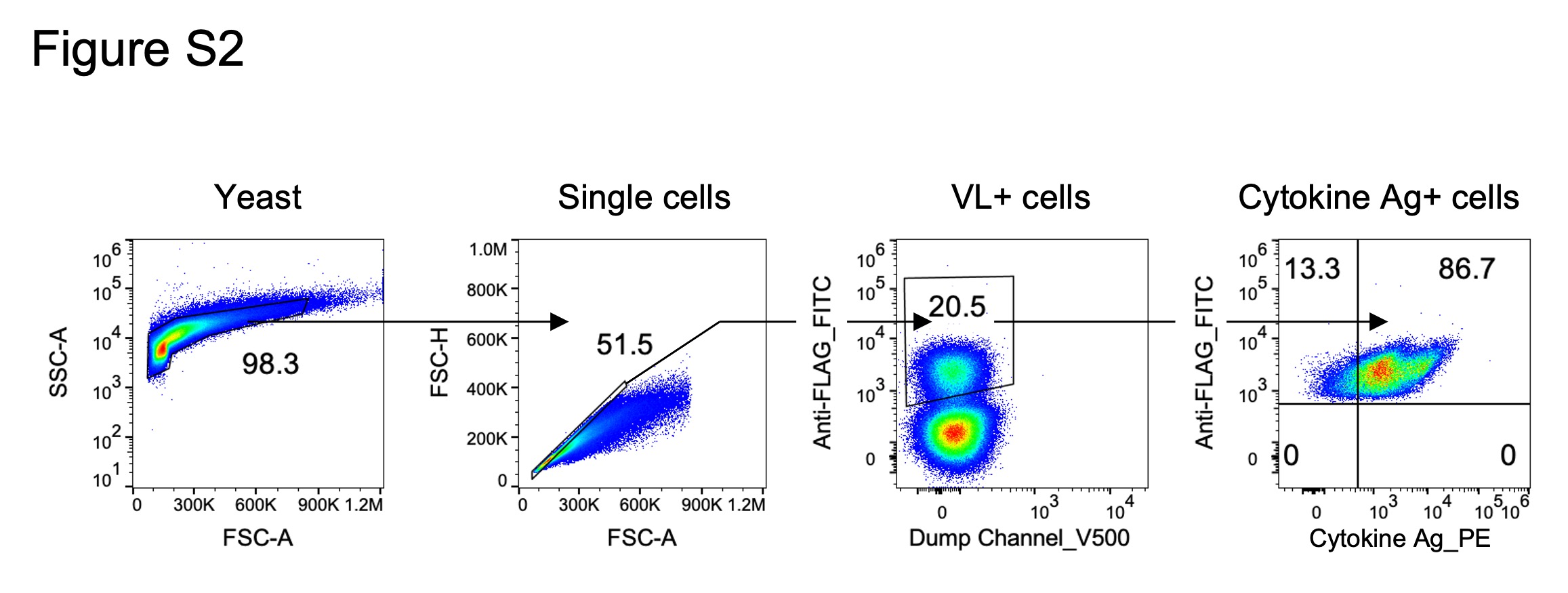

Supplement: Supplementary Figure 2 — Representative FACS gating strategy for yeast display functional screening. Yeast cells were stained with anti-FLAG_FITC and cytokine_PE for sorting. [file Image_2.jpg]

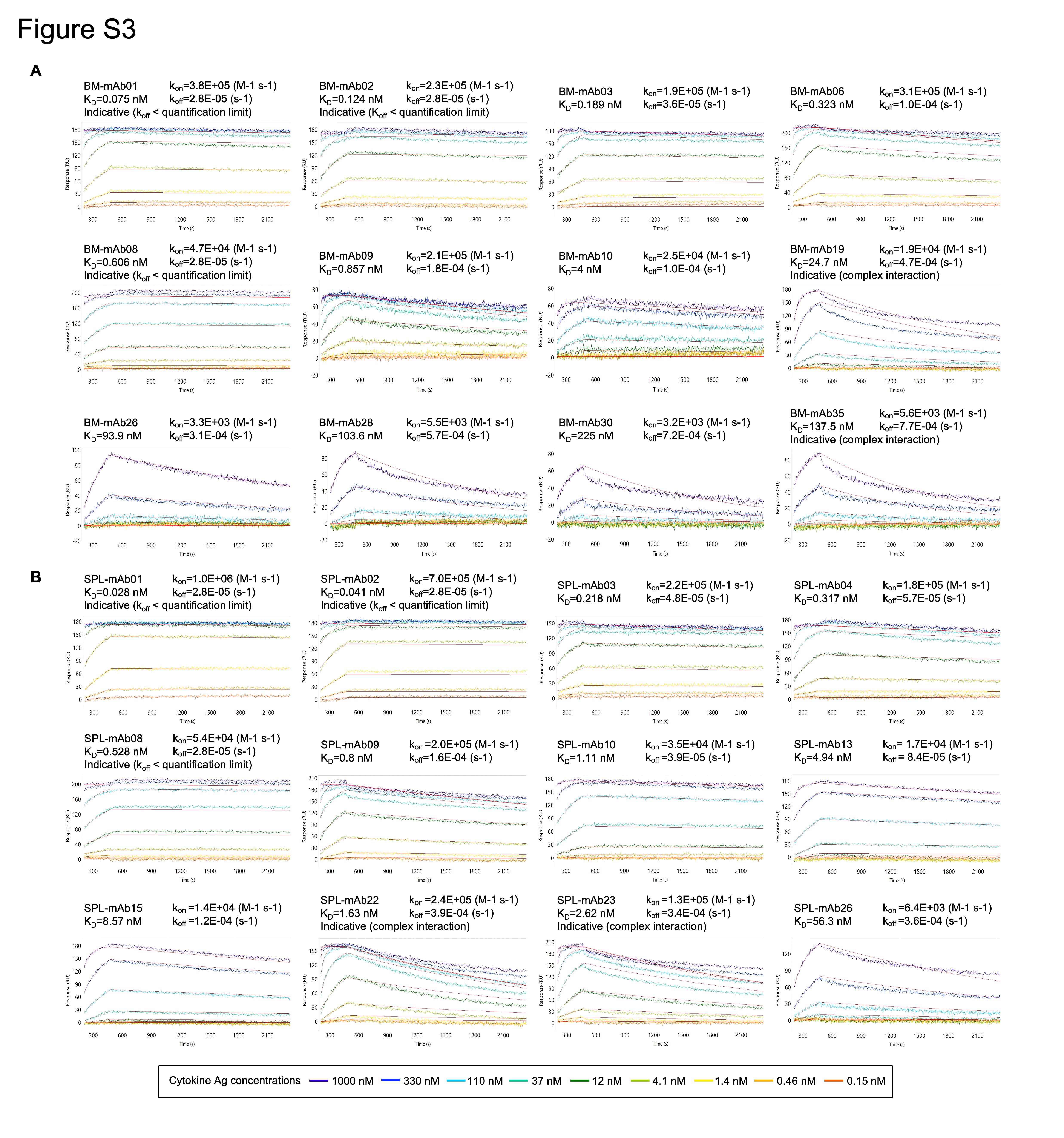

Supplement: Supplementary Figure 3 — Representative SPR sensorgrams for antibody binding to cytokine protein. (A) Representative mAbs from the bone marrow IgG library. (B) Representative mAbs from the spleen IgG library. [file Image_3.jpg]

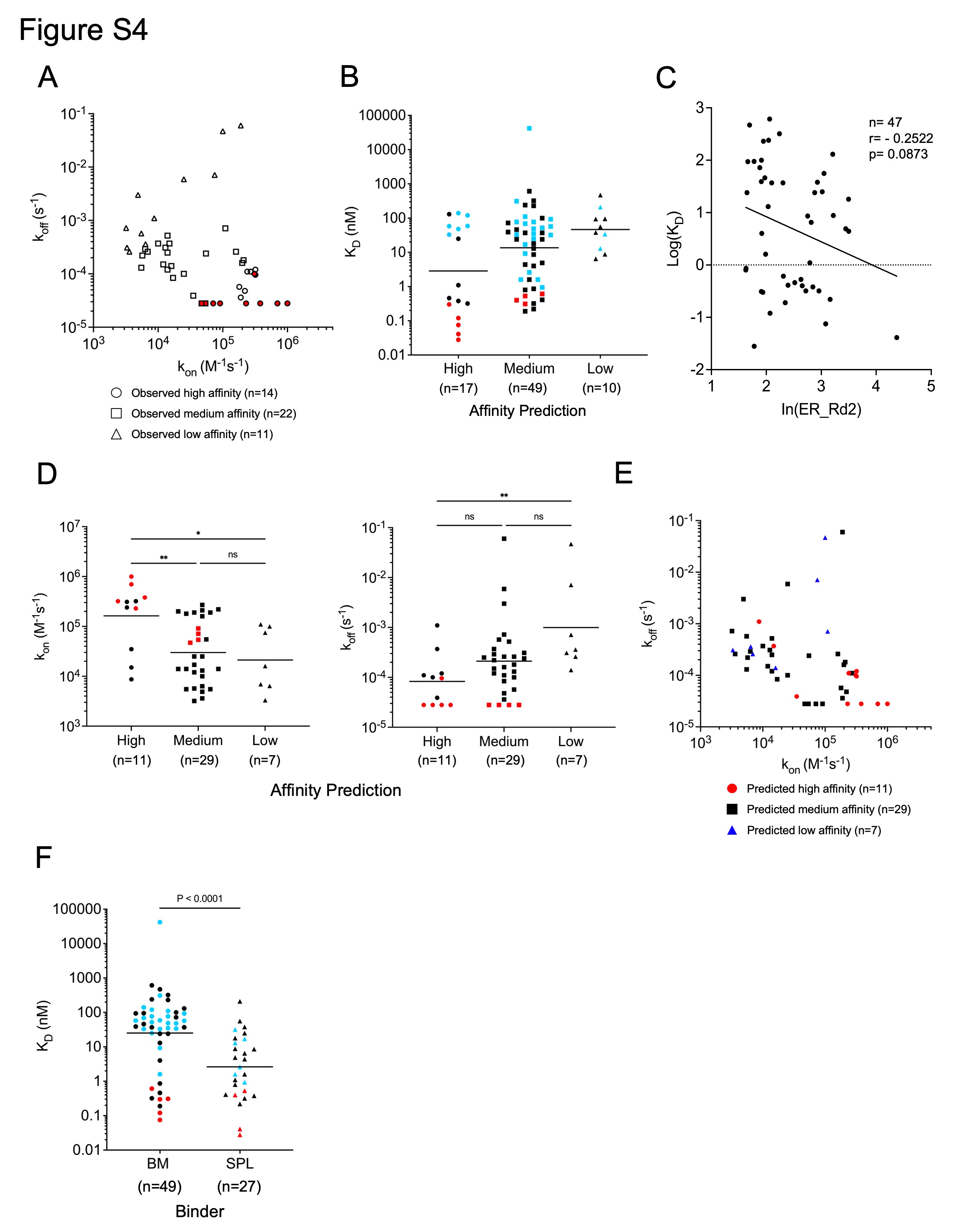

Supplement: Supplementary Figure 4 — Predicted affinity vs. observed affinity analyses. (A) A scatter plot of kon vs. koff for genuine antibody binders; antibodies with complex interactions by SPR were omitted, and points were categorized by observed affinity. Red points represent the clones that had koff below the quantification limit. (B) Correlation between predicted affinity and observed affinity for all binding antibodies tested. Each point represents a single binder. Red points represent the clones that had koff below the quantification limit. Blue points represent the clones that had complex interactions, which were omitted from Figure 3C. Bar represents the geometric mean value of the group. No statistical significance was observed across each affinity group by a one-way ANOVA (Kruskal-Wallis) test. (C) Correlation between Round 2 ER and observed affinity (KD) for genuine antibody binders; antibodies with complex interactions by SPR were omitted. Pearson correlation coefficient r and p value are shown in the plot. (D) Correlation between predicted affinity and observed kon (left) and koff (right) for genuine antibody binders; antibodies with complex interactions by SPR were omitted. Red points represent the clones that had koff below the quantification limit. Bar represents the geometric mean value of the group. A one-way ANOVA (Kruskal-Wallis) test was used to determine statistical significance (**p< 0.01, *p< 0.05). (E) A scatter plot of kon and koff for genuine antibody binders; antibodies with complex interactions by SPR were omitted, and points were categorized by predicted affinity. Red points represent the clones that had koff below the quantification limit. (F) Comparison of observed affinity between all bone marrow and spleen binding antibodies. Red points represent the clones that had koff below the quantification limit. Blue points represent the clones that had complex interactions, which were omitted from Figure 3D. Bar represents the geometric mean value of the group. Statistical s [file Image_4.jpg]

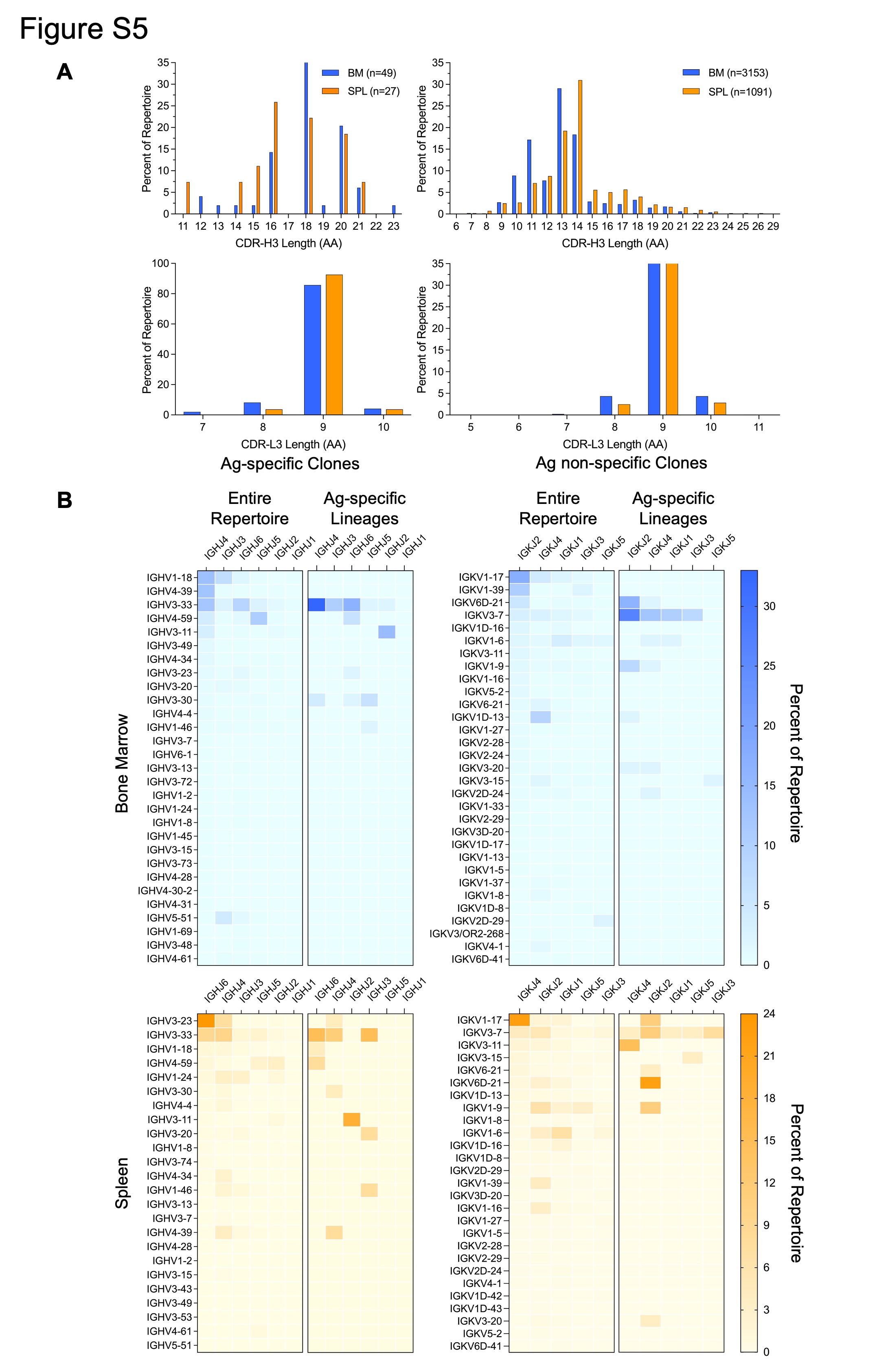

Supplement: Supplementary Figure 5 — Genetic analysis of bone marrow and spleen antibody repertoires. (A) CDR-H3 (upper) and CDR-L3 (lower) amino acid length distributions for the SPR-confirmed antigen-specific clones (right) and the rest of the repertoires (left) of bone marrow and spleen libraries. (B) IGHV/IGHJ and IGKV/IGKJ gene usage for the SPR-confirmed antigen-specific lineages and overall antibody repertoires of bone marrow and spleen libraries. [file Image_5.jpg]

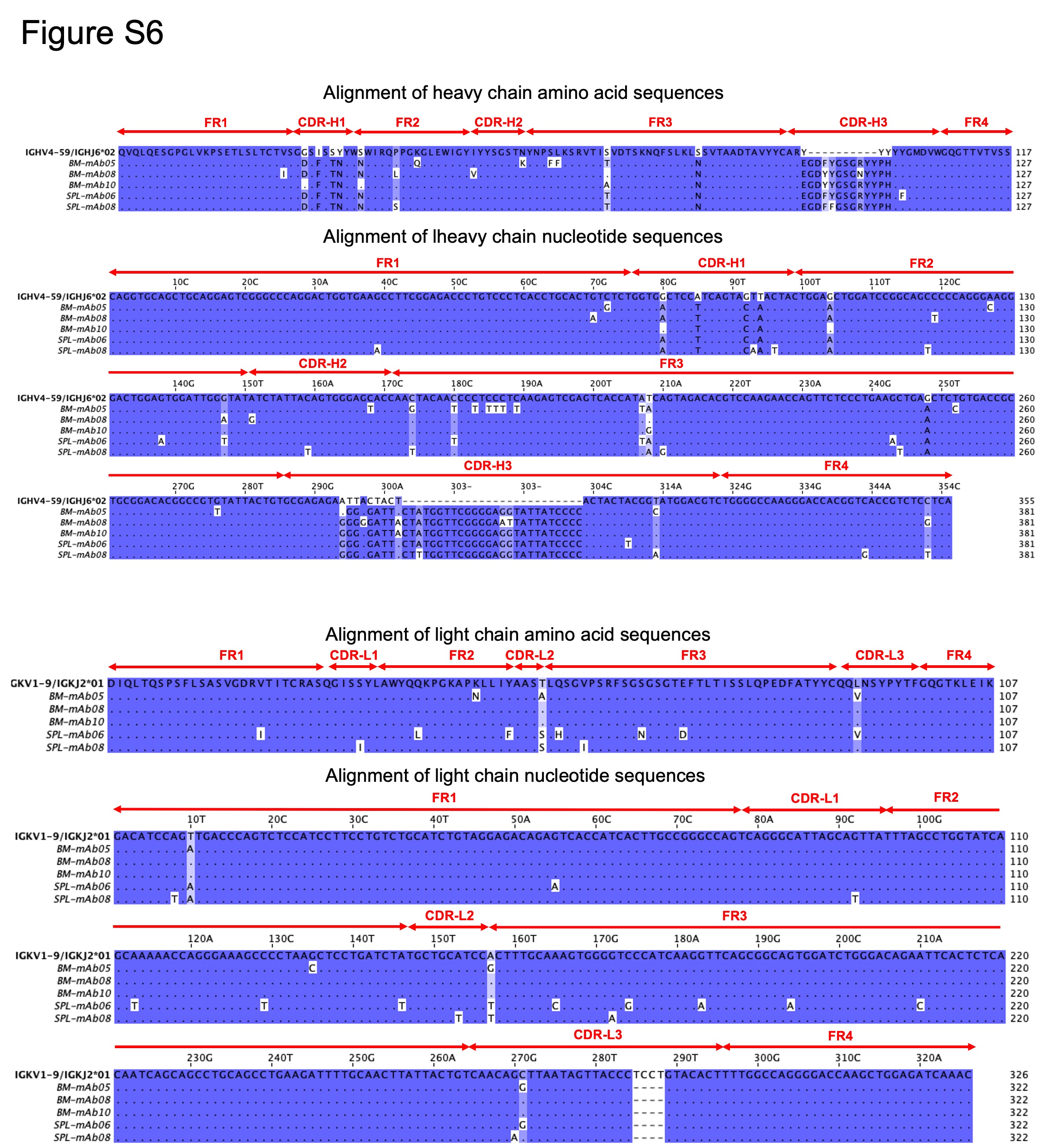

Supplement: Supplementary Figure 6 — Representative multiple sequence alignments of antibodies from bone marrow and spleen libraries that shared the same gene usage of IGHV4-59/IGHJ6*02_IGKV1-9/IGKJ2*01. Alignments were shown for both heavy chain and light chain amino acid sequences and nucleotide sequences. [file Image_6.jpg]
